# Urinary IgG and ****α****2-Macroglobulin Are Powerful Predictors of Outcome and Responsiveness to Steroids and Cyclophosphamide in Idiopathic Focal Segmental Glomerulosclerosis with Nephrotic Syndrome

**DOI:** 10.1155/2013/941831

**Published:** 2013-09-04

**Authors:** Claudio Bazzi, Virginia Rizza, Daniela Casellato, Gilda Stivali, Gregorio Rachele, Pietro Napodano, Maurizio Gallieni, Giuseppe D'Amico

**Affiliations:** ^1^D'Amico Foundation for Renal Diseases Research, Via Cherubini, 6, 20100 Milan, Italy; ^2^Biochemical Laboratory, San Carlo Borromeo Hospital, 20147 Milan, Italy; ^3^Nephrology and Dialysis Unit, San Carlo Borromeo Hospital, 20147 Milan, Italy; ^4^Baxter Renal, 00144 Rome, Italy

## Abstract

*Objective. *To assess whether high-molecular-weight proteins excretion predicts outcome and therapy-responsiveness in patients with FSGS and nephrotic syndrome. *Research Design and Methods*. Thirty-eight patients measured at biopsy fractional excretion of IgG (FEIgG) and urinary **α**2-macroglobulin/creatinine ratio (**α**2m/C). Low and high risk groups were defined by cutoffs assessed by ROC analysis. In all patients first-line therapy was with steroids alone or in combination with cyclophosphamide. *Results.* **α**2m/C and FEIgG were correlated with segmental sclerosis (*r* = 0.546; *r* = 0.522). Twenty-three patients (61%) entered Remission and 9 (24%) progressed to ESRD. Comparing low and high risk groups, by univariate analysis remission was predicted by FEIgG (77% versus 25%, *P* = 0.016) and **α**2m/C (81% versus 17%, *P* = 0.007) and ESRD at best by FEIgG (0% versus 75%, *P* < 0.0001) and **α**2m/C (4% versus 67%, *P* < 0.0001). By multivariate analysis FEIgG was the only independent predictor of remission and **α**2m/C the most powerful predictor of ESRD. Low and high risk groups of FEIgG and **α**2m/C in combination had very high predictive value of sustained remission and ESRD in response to therapy. *Conclusions. *FEIgG and **α**2m/C are powerful predictors of outcome and responsiveness to steroids and cyclophosphamide; their predictive value, if validated in prospective studies, may be useful in clinical practice suggesting first-line alternative treatments in high risk patients.

## 1. Introduction

 Idiopathic focal segmental glomerulosclerosis (FSGS) is a clinicopathologic entity characterized by alteration of the molecular architecture of podocytes and slit diaphragms with disruption of the glomerular filtration barrier (GFB) and consequent proteinuria. Studies of animal models and familiar forms elucidated several molecular defects responsible for podocyte damage [1–3]. An increased understanding of the molecular mechanisms involved in podocyte damage has not been associated as yet with improved outcome prediction [2]; thus at present the best favourable prognostic factor for FSGS with nephrotic syndrome (NS) is still remission in response to corticosteroids [4–6]. The etiology of idiopathic FSGS is unknown. An immunological pathogenesis has been hypothesized, at least in some patients, with a clone of T lymphocytes secreting a permeability factor that alters GFB [7]. Recently some doubts have been raised regarding the autoimmune pathogenesis of FSGS (review in [8]), supported in part by the observation that certain “immunosuppressive” agents (dexamethasone and cyclosporine A) reduce proteinuria by a direct stabilization of the podocyte cytoskeleton [9–12]. On the basis of the immunologic hypothesis, idiopathic FSGS has been treated over time with older and more recent immunosuppressive agents, but treatment is still largely empirical due to different and unpredictable responses and the lack of reliable predictors for drug responsiveness. As early as 1976, Hardwicke et al [13]. showed that FSGS is characterized by elevated excretion of the high-molecular-weight (HMW) protein IgG. In a pilot observational study [14], we showed that baseline fractional excretion of IgG (FE IgG) is a reliable predictor of ESRD and remission and that responsiveness to steroids alone or steroids plus cyclophosphamide is dependent on the value of FE IgG. The aim of the study was to evaluate in a long-term observational study the predictive value of functional outcome and responsiveness to steroids alone or in combination with cyclophosphamide of baseline excretion of HMW proteins (IgG, 150 kDa; *α*2-macroglobulin, 720 kDa) as markers of selectivity of GFB [15].

## 2. Subjects and Methods

### 2.1. Patients

 The present study is a follow-up of our previous study [14]; the number of patients has increased from 29 to 38 with inclusion of 9 patients diagnosed after the publication of the previous study; all patients are white Europeans. Inclusion criteria are presence of NS defined as 24 hour proteinuria ≥3.5 g; at least 6 glomeruli in biopsy specimens; measurement at biopsy of urinary *α*2-macroglobulin/creatinine ratio (*α*2m/C), FE IgG, and fractional excretion of *α*1-microglobulin (FE *α*1m), 24 hour proteinuria and urinary protein/creatinine ratio; baseline sCr <2.0 mg/dL and eGFR ≥30 mL/min/1.73 m^2^; follow-up of at least 24 months in patients not progressing to ESRD; overall follow-up: 96 ± 67 months (12–234); and follow-up of patients not progressing to ESRD: 115 ± 63 months (24–234). The study is in adherence to the Declaration of Helsinki. All patients gave informed consent to data handling. The clinical features of patients are reported in Table 1.

### 2.2. Renal Biopsies

 Renal biopsies were performed by standard histologic and immunofluorescence methods [16]; 35 biopsies were available for analysis and were evaluated by the pathologist P. N.: the types of histologic variants were defined according to the Columbia classification [17]: NOS 71% (*n* = 25), cellular 23% (*n* = 8), tip 3% (*n* = 1), and perihilar 3% (*n* = 1). The mean number (±SD) of glomeruli in biopsies was 14 ± 6 (6–32). The percentages of glomeruli with global glomerulosclerosis (GGS) and focal segmental glomerulosclerosis (SS) were 7 ± 10% (0–37%) and 21 ± 15% (5–70%), respectively. The extent of tubulointerstitial damage (TID) was evaluated semiquantitatively: tubular atrophy, interstitial fibrosis, and inflammatory cell infiltration were graded 0, 1, or 2 if absent, focal, or diffuse (TID global score: 0–6); mean (±SD) of TID score: 2.0 ± 1.5 (0–6). 

### 2.3. Laboratory Analysis

 For each patient a 24 hour urine collection and a second morning urine sample were obtained at biopsy. Urinary proteins were measured by the Coomassie blue method and expressed in grams/24 hours (24 hP) and as protein/creatinine ratio (UP/C: mg/1g of urinary creatinine). Serum and urinary creatinine were measured with standard automated techniques. Baseline and last eGFR were calculated according to the 4-variable MDRD formula [18]. IgG, *α*1-microglobulin (*α*1m), and *α*2-macroglobulin (not evaluated in the previous study) [14] were measured by immunonephelometry as described [14]. FE IgG and FE *α*1m were calculated according to the formula (urinary protein/serum protein × sCr/uCr) × 100; *α*2m/C was expressed in mg/1 g urinary creatinine.

### 2.4. End Points

 Two end points were considered: (1) ESRD with start of renal replacement therapy and (2) remission: complete (24 hP < 0.2 g/day) or partial (24 hP < 2.0 g/day) with stable renal function.

### 2.5. Predictors of Functional Outcome

 Nine factors were evaluated for their predictive value of outcome: eGFR, *α*2m/C, FE IgG, FE a1m, 24 hP, UP/C, GGS, TID score and SS. For each parameter the cutoff point with the highest sensitivity and specificity for progression to ESRD assessed by ROC analysis was used to define low and high risk groups.

### 2.6. Treatment

All 38 patients started treatment with steroids soon after biopsy: 26 of them (68%) with 3 i.v. methylprednisolone pulses (1 g/day on alternate days) followed by oral prednisone (1 mg/kg/day with tapering) and 12 patients (32%) started treatment with oral prednisone (1 mg/kg/day with tapering). The duration of oral treatment with prednisone in all 38 patients was 9.3 ± 5.5 months [4–32]. Steroid responsiveness (complete or partial remission) was assessed at the end of the fourth month of steroid treatment; 13 patients (34%) were steroid responsive; 25 patients (66%) were unresponsive; in 28 patients (20 steroid unresponsive and 8 steroid responsive but relapsing) treatment with cyclophosphamide (CYP) was associated with steroids: in 10 patients with i.v. monthly pulses of 0.5–0.75 g for 3–6 months and in 18 patients with oral CYP (1.5–2.0 mg/kg/day for 3.7 ± 1.6 months (2–6.5); lower doses for elderly patients and patients with renal function impairment.

### 2.7. Statistical Methods

 The SPSS18 software program was used for statistical analysis. Differences between groups were determined using the unpaired *t*-test and the Mann-Whitney *U* test. Correlations were assessed with the Spearman test. The receiver operating characteristics (ROC) curve was used to determine cutoff values for progression to ESRD of functional, proteinuric, and histologic parameters. For the end point ESRD and remission survival curves according to Kaplan-Meier were used to evaluate differences between low and high risk groups; equality of survival curves was tested by log-rank test. Multivariate Cox regression analysis identified the independent predictors of ESRD and remission. The significance level was defined as *P* < 0.05.

## 3. Results

### 3.1. Correlation between Histologic Lesions and Proteinuric Markers

The percentage of SS was highly correlated with *α*2m/C (*r* = 0.546, *P* = 0.001), FE IgG (*r* = 0.522, *P* = 0.001) and FE *α*1m (*r* = 0.373, *P* = 0.027), but not with 24 hP and UP/C. The patients with SS below versus above the SS median value (16%) had significantly different levels of *α*2m/C (0.5 ± 1.4 versus 5.7 ± 6.1, *P* < 0.0001), FE IgG (0.038 ± 0.043 versus 0.116 ± 0.102, *P* = 0.001), and FE *α*1m (0.177 ± 0.117 versus 0.320 ± 0.178, *P* = 0.009), while the difference was not significant for 24 hP and UP/C. Other chronic lesions (GGS and TID scores) did not show a significant correlation with all proteinuric markers.

### 3.2. Correlation between Baseline Functional and Proteinuric Parameters and Last eGFR

The eGFR at last observation was highly correlated with baseline eGFR (*r* = 0.502, *P* = 0.001), *α*2m/C (*r* = −0.546, *P* < 0.0001), FE IgG (*r* = −0.565, *P* < 0.0001), and FE *α*1m (*r* = −0.563, *P* < 0.0001); lower degree of correlation was found for 24 hP (*r* = −0.357, *P* = 0.028) and UP/C (*r* = −0.394, *P* = 0.014).

### 3.3. ROC Analysis for Progression to ESRD, Sensitivity and Specificity of Cutoffs

**Figure 1:**
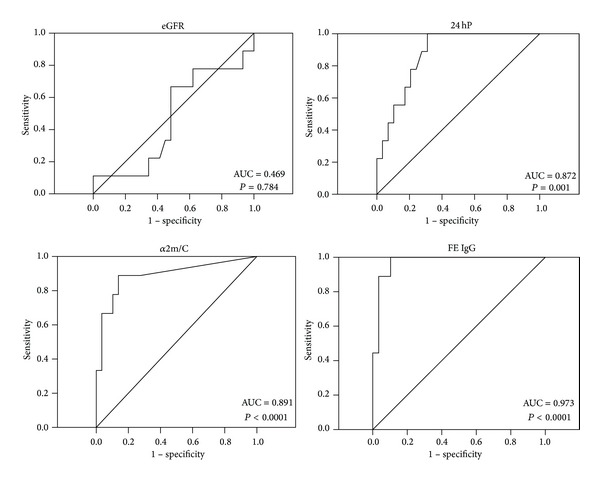
Area under the ROC curves (AUC) for progression to ESRD of the parameters eGFR, 24 hour proteinuria (24 hP), fractional excretion of IgG (FE IgG), and urinary *α*2-macroglobulin/creatinine ratio (*α*2m/C).

To evaluate the predictive value of functional outcome low and high risk groups were defined for all parameters according to cutoffs with the highest sensitivity and specificity for progression to ESRD assessed by ROC analysis (Table 2 and Figure 1). FE IgG showed the largest area under the ROC curve: 0.973; cutoff ≥0.112; sensitivity: 100%; specificity: 90%.

### 3.4. Overall Functional Outcome

 Nine patients (24%) reached ESRD and started renal replacement therapy after 34 ± 35 months (12–119); 23 patients (61%) entered remission after 15 ± 19 months (range 1–75); 6 patients (16%) had persistent NS with stable eGFR after 60 ± 29 months (24–73): baseline versus last eGFR: 63 ± 25 versus 60 ± 36 mL/min/1.73 m^2^, *P* = ns. 

The ESRD patients compared to remission patients had significantly higher values of 24 hP, UP/C, *α*2m/C, FE IgG, FE *α*1m and SS (Table 3), no significant differences for age, baseline eGFR, percentage of eGFR <60 mL/min, high baseline blood pressure, GGS and TID scores.

### 3.5. Prediction of Remission as First Event after NS

**Figure 2:**
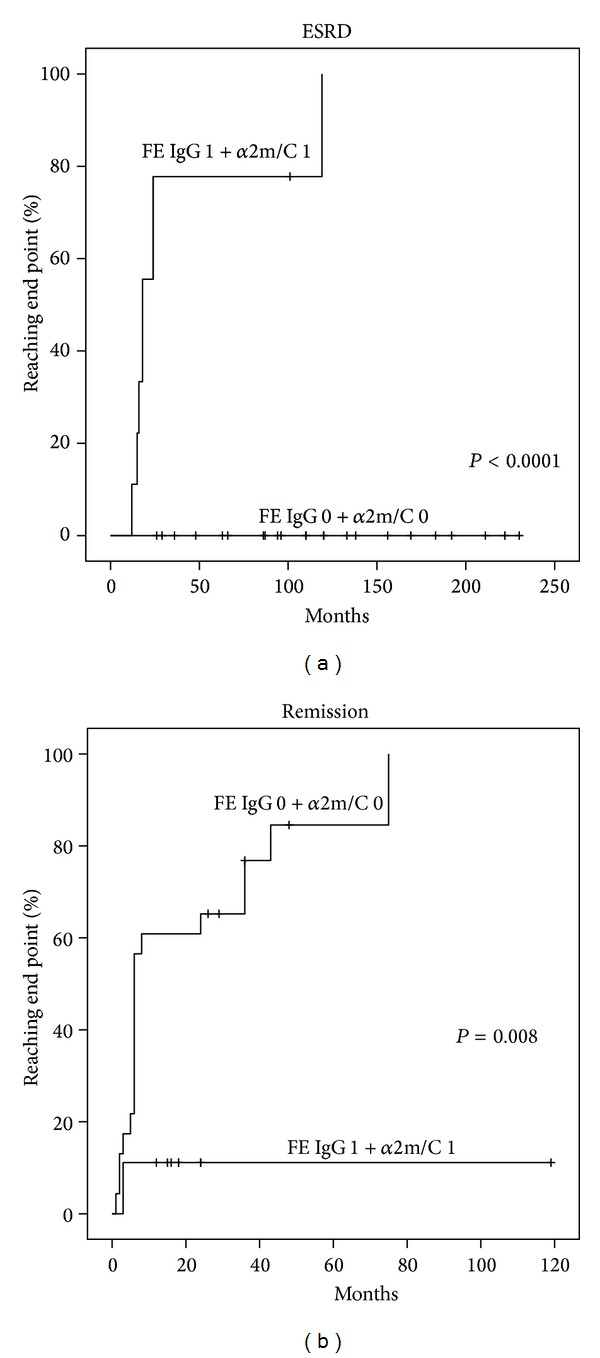
Probability of ESRD and remission in patients with FE IgG and *α*2m/C below (0) or above (1) their respective cutoffs.

 By univariate analysis only FE IgG (77% versus 25%, *P* = 0.016) and *α*2m/C (81% versus 17%, *P* = 0.007) were predictors of remission in patients with a value below or above their cutoff (Table 4). By multivariate analysis according to Cox model including FE IgG and *α*2m/C, the independent predictor of remission was FE IgG (HR: 0.18, CI 0.041–0.753, *P* = 0.019). Prediction of remission increased to 83% versus 11% (*P* = 0.008) in patients with both FE IgG and *α*2m/C below or above their respective cutoffs (Table 4 and Figure 2).

### 3.6. Relationship between Remission and Type of Treatment

 All 23 patients who entered remission started therapy soon after renal biopsy with steroids; 13 patients (34%) were steroid responsive and entered remission within 4 months, and 10 patients were steroid unresponsive with persistent NS at the end of fourth month, when CYP treatment was associated: 8 of them (21%) entered remission; the overall remission rate in response to steroids or steroids + cyclophosphamide was 55%. Two patients were unresponsive to steroids and CYP in combination: one entered remission after treatment with mycophenolate mofetil (2 g/day for 18 months) and the other after treatment with 400 mg × 2/day for 43 months of pentoxifylline, a non-specific phosphodiesterase inhibitor with anti-inflammatory properties [19, 20] which reduces proteinuria in membranous [21] and diabetic nephropathy [22] and slows the GFR decline in CKD [19]. Ten of the 13 steroid-responsive patients had 13 relapses; 2 of them entered sustained remission after a second treatment with steroids alone and 5 patients after one or more treatments with steroids plus CYP; 3 patients became steroid dependent; of these one patient entered remission after cyclosporine A (200 mg/day with tapering for 32 months); one patients was unresponsive to treatment with rituximab (600 mg × 2, 18 months apart) and one unresponsive to treatment with mycophenolate mofetil (2 g/day for 12 months). 

### 3.7. Sustained Remission

At last observation after 138 ± 56 months, 21 out of 23 patients with remission as first event after NS had sustained remission for 104 ± 54 months with steroids alone (*n* = 5), steroids + CYP (*n* = 13), or other treatments in 3 patients unresponsive to steroids and CYP (*n* = 2) or steroid dependent (*n* = 1): mycophenolate mofetil (no. 1), pentoxifylline (*n* = 1), and cyclosporine A (*n* = 1), respectively; 18 (86%) out of 21 patients who attained sustained remission had FE IgG and *α*2m/C below the cutoff. Remission was complete in 13 patients and partial in 8 patients; baseline versus last values of eGFR and 24 hP were 83 ± 31 versus 80 ± 23 mL/min/1.73 m^2^ (*P* = ns) and 7.3 ± 3.1 versus 0.28 ± 0.39 g/24 hours, *P* < 0.0001, respectively. 

### 3.8. ESRD Prediction

By univariate analysis, the most powerful predictors of ESRD were FE IgG (0% versus 75% *P* < 0.0001), *α*2m/C (4% versus 67%, *P* < 0.0001), and FE *α*1m (7% versus 70%, *P* < 0.0001) in patients with baseline value below or above their cutoff. All other functional, proteinuric, and histologic markers had lower predictive value (Table 4). By multivariate analysis according to Cox model including UP/C, FE IgG, *α*2m/C, and FE *α*1m, the most powerful independent predictors of ESRD were *α*2m/C (HR: 16, CI 1.8–142, *P* = 0.013) and FE*α*1m (HR: 5.9, CI 1.1–31.3, *P* = 0.038). ESRD prediction increased to 0% versus 89% (*P* < 0.0001) in patients with both FE IgG and *α*2m/C below or above their respective cutoffs (Table 4 and Figure 2).

### 3.9. Relationship between ESRD, Proteinuric Markers, and Type of Treatment

The 9 ESRD patients started steroid therapy soon after biopsy: 8 with 3 i.v. methylprednisolone pulses (1 g/day on alternate days) followed by oral prednisone 1 mg/kg/day with tapering for 9 ± 3 months (5–12); all patients were steroid unresponsive at the end of the fourth month; in 8 patients cyclophosphamide treatment was associated: in 4 patients with 0.5–0.75 g iv pulses monthly for 3–6 months and in 4 patients with 1.5–2.0 mg/day for 2.5 ± 1.0 months (2–4). All 9 ESRD patients were steroid unresponsive and 8 steroid + CYP unresponsive; in all patients FE IgG was above the cutoff.

### 3.10. Relationship between FE IgG and Responsiveness to Steroids Alone or Steroids Plus Cyclophosphamide

FE IgG was significantly lower in patients responsive to steroids alone versus patients responsive only to steroids plus CYP (0.034 ± 0.047 versus 0.084 ± 0.038, *P* = 0.008) and versus patients unresponsive to steroids plus CYP and progressing to ESRD (0.034 ± 0.047 versus 0.234 ± 0.144, *P* < 0.0001); FE IgG was also lower in patients responsive only to steroids plus CYP versus patients unresponsive to steroids plus CYP (0.084 ± 0.038 versus 0.234 ± 0.144, *P* = 0.008). 

## 4. Discussion 

Our data show that the excretion of HMW proteins (*α*2m and IgG) is highly correlated with SS (*r* = 0.546 and 0.522, resp.); this observation suggests that development and percentage of SS are associated with loss of selectivity of GFB of which FE IgG and *α*2m/C are reliable markers [15]. By contrast all proteinuric markers did not correlate with the chronic lesions GGS and TID scores. This lack of correlation may be dependent on time of biopsy in relation to the onset of NS; 55% of our patients had biopsies within 4 months after the onset of NS (37% within 2 months), a time that may be too short for the development of chronic lesions, while SS develops early in the disease course as is present in 15 ± 11% versus 29 ± 17% of glomeruli (*P* = 0.017) in patients biopsied within 4 months or later. 

FE IgG and *α*2m/C show the highest prediction of functional outcome; by univariate analysis their prediction of ESRD and remission is higher than that of all other markers, including 24 hP, the most widely used marker of NS severity. By multivariate analysis FE IgG is the only independent predictor of remission and *α*2m/C the most powerful independent predictor of ESRD. These data suggest that the more severe the GFB alteration and the more elevated the excretion of HMW proteins, the higher the risk of progressive renal damage, mediated at least in part by TID, as suggested by the high correlation between FE IgG and FE *α*1m (*r* = 0.759, *P* < 0.0001) and between FE *α*1m and last eGFR (*r* = − 0.563). Conversely the lower the excretion of FE IgG and *α*2m/C, the higher the probability of remission.

A high predictive value of outcome of HMW protein excretion has been observed in other types of GN; IgG in glomerulonephritis [23, 24], idiopathic membranous nephropathy [25–27], crescentic [28] and noncrescentic IgA nephropathy [29, 30] with different cutoffs for each type of GN; IgM (MW: 900 kDa) in ANCA-associated renal vasculitis [31] and type 2 diabetic nephropathy [32]. 

FE IgG is also a predictor of responsiveness to steroids alone or in combination with CYP; it is significantly lower in patients responsive to steroids alone versus patients responsive only to steroids and CYP in combination (*P* = 0.008) and versus patients unresponsive to both drugs and progressing to ESRD (*P* < 0.0001). 

Progression to ESRD in patients treated with steroids + CYP according to FE IgG and *α*2m/C below or above their cutoff is 0% versus 89% (*P* < 0.0001), suggesting that this type of therapy prevents progression in all patients with low risk profile. Sustained remission, assessed according to FE IgG and *α*2m/C in combination, is 83% versus 11% (*P* = 0.008), suggesting that low risk profile is associated with a very high percentage of sustained remission. The present study in comparison with our previous study [14] includes some interesting new data: (1) the excretion of HMW protein *α*2-macroglobulin, strongly correlated with the percentage of SS, in combination with FE IgG, increases the prediction of remission in low risk patients (83%) and progression to ESRD in high risk patients (89%). (2) The long-term follow-up of patients not progressing to ESRD shows that baseline low FE IgG and *α*2m/C below their cutoff are long-term predictors of sustained remission: 21 out of 23 patients who attained remission as first event after NS had sustained remission (follow-up 138 ± 56 months); 18 of these patients had FE IgG and *α*2m/C below the cutoff: 15 treated with steroids alone (*n* = 4) or in combination with CYP (*n* = 11) and 3, unresponsive to steroids plus CYP (*n* = 2) or steroid dependent (*n* = 1), after treatment with mycophenolate mofetil (*n* = 1), pentoxifylline (*n* = 1), and cyclosporine A (*n* = 1). Thus a mild baseline alteration of GFB suggested by low FE IgG and *α*2m/C is a long-term predictor of sustained remission and shows that responsiveness to steroids plus CYP is less disappointing than usually stated [33] if evaluated in low risk patients since 11 out of 18 low risk patients (61%) are responsive to steroids plus CYP. (3) The prediction of progression to ESRD by high FE IgG and *α*2m with baseline value below or above their cutoff (0% versus 89%) suggests that this type of therapy prevents progression only in low risk patients.

Thus the availability of baseline biomarkers able to evaluate risk profiles and responsiveness to steroids alone or in combination with cyclophosphamide may improve clinical practice; if the predictive value of outcome and responsiveness to steroids alone or in combination with CYP of these biomarkers can be validated in prospective studies, the usual approach to treatment may change suggesting a first-line therapy with alternative agents in high risk patients soon after biopsy and not later in the course of disease when some degree of progressive chronic damage may have occurred. Early start of therapy after disease onset is very important; in our patients with FE IgG and *α*2m/C below the cutoff remission rate was 93% versus 60% (*P* = 0.047) in patients biopsied within four months after the onset of NS or later.

For the treatment of patients unresponsive to steroids alone or associated with alkylating agents, several other more or less recent agents, have been used (review in [2]): calcineurin inhibitors (cyclosporine A, tacrolimus), mycophenolate mofetil, monoclonal antibodies (rituximab, adalimumab), rosiglitazone, and galactose with variable and unpredictable results due to a lack of reliable outcome predictors. It is reasonable to assume that FE IgG and *α*2m/C might have a predictive value for responsiveness also to these agents; a recent study [34] of idiopathic membranous nephropathy showed that FE IgG predicts remission at 12 months in patients treated with rituximab.

The main limitation of this study is its long-term uncontrolled observational design in a rather small group of patients with idiopathic FSGS. It should be taken into account that FSGS is a rather uncommon disease and the suggestion that both well-conducted observational studies and randomized controlled trials play a complementary and valuable role in renal diseases [35]. 

In conclusion this study identifies baseline biomarkers able to evaluate the degree of GFB alteration and predict functional outcome and responsiveness to steroids and CYP. The ability of these biomarkers to identify at baseline patients unresponsive to steroids and CYP, if validated in prospective studies, may improve clinical practice, suggesting the choice of first-line alternative treatments which may be more successful if started early in the disease course. A validation of the predictive value of outcome and responsiveness to new drugs of these biomarkers in large patient cohorts is warranted.

## Figures and Tables

**Table 1:** Baseline clinical characteristics of 38 patients with FSGS and NS.

No. of patients	38	Range
Age (yrs)	39 ± 18	14–80
Sex (M/F)	21/17	
eGFR (mL/min/1.73 m^2^)	80 ± 31	30–123
eGFR <60 mL/min/1.73 m^2^	29%	
BP ≥140/90 mmHg	55%	
Serum albumin	2.26 ± 0.72	0.98–3.76
24 hP	8.5 ± 5.7	3.5–32.7
UP/C	6111 ± 4246	253–20283
*α*2m/C	3.4 ± 5.2	0–18.7
FE IgG	0.091 ± 0.112	0.003–0.534
FE *α*1m	0.305 ± 0.260	0.007–0.945
No. of glomeruli in RB (no. 35)	14 ± 6	6–32
GGS%	7 ± 10	0–37
TID score	2.0 ± 1.5	0–6
SS%	21 ± 15	5–70
Overall follow-up (mths)	96 ± 67	12–236
Follow-up of pts. without ESRD	115 ± 63	24–236

eGFR: estimated GFR; BP: blood pressure; 24 hP: 24 hour proteinuria; UP/C: urinary protein/creatinine ratio; *α*2m/C: urinary *α*2m/creatinine ratio; FE IgG: fractional excretion of IgG; FE *α*1m: fractional excretion of *α*1-microglobulin; RB: renal biopsy; GGS: global glomerular sclerosis; TID score: tubulointerstitial damage score; SS: segmental sclerosis.

**Table 2:** Area under the ROC curve (AUC), cutoffs, sensitivity, and specificity for progression to ESRD of clinical, proteinuric, and histological parameters.

Risk factors	AUC	*P*	Cutoff	Sensitivity %	Specificity %
eGFR	0.489	(0.784)	≥68	67	52
FE IgG	0.973	(<0.0001)	≥0.112	100	90
UP/C	0.904	(<0.0001)	≥5980	100	76
FE *α*1m	0.897	(<0.0001)	≥0.362	78	93
*α*2m/C	0.891	(<0.0001)	≥4.79	89	86
24 hP	0.872	(0.001)	≥6.8	100	69
GGS	0.470	(0.798)	≥7.5%	38%	59%
TID score	0.477	(0.844)	≥3.5	25%	93%
SS	0.701	(0.087)	>16%	88%	59%

eGFR: estimated GFR; FE IgG: fractional excretion of IgG; UP/C: urinary protein/creatinine ratio; FE *α*1m: fractional excretion of *α*1-microglobulin; *α*2m/C: urinary *α*2-macroglobulin/creatinine ratio; 24 hP: 24 hour proteinuria; GGS: global glomerular sclerosis; TID score: tubulointerstitial damage score; SS: segmental sclerosis.

**Table 3:** Clinical, proteinuric, and histological parameters in patients who progressed to ESRD compared to patients who entered remission as first event.

	ESRD	Remission	*P*
No. of patients	9	23	
Age (yrs)	33 ± 21	41 ± 17	ns
Sex (M/F)	6/3	14/9	ns
Baseline eGFR mL/min/1.73 m^2^	76 ± 30	85 ± 31	0.46
eGFR <60 mL/min/1.73 m^2^	22%	26%	ns
BP ≥140/90 mmHg	56%	57%	ns
24 hP	14.1 ± 8.6	7.2 ± 3.0	0.003
UP/C	10486 ± 4100	5125 ± 3581	0.001
*α*2m/C	9.5 ± 6.4	1.2 ± 2.1	<0.001
FE IgG	0.234 ± 0.144	0.051 ± 0.050	<0.001
FE *α*1m	0.485 ± 0.204	0.210 ± 0.139	<0.001
Segmental sclerosis %	26 ± 13	18 ± 15	0.043
Global glom. sclerosis %	7 ± 13	5 ± 7	0.91
TID score	2.0 ± 2.1	1.6 ± 1.3	0.85
Time to ESRD (mths)	34 ± 35		
Time to first remission (mths)		15 ± 19	

eGFR: estimated GFR; BP: blood pressure; 24 hP: 24 hour proteinuria; UP/C: urinary protein/creatinine ratio; *α*2m/C: urinary *α*2-macroglobulin/creatinine ratio; FE IgG: fractional excretion of IgG; FE *α*1m: fractional excretion of *α*1-microglobulin; GGS: global glomerular sclerosis; TID score: tubulointerstitial damage score; SS: segmental sclerosis.

**Table 4:** ESRD and remission rate in 38 patients with FSGS and NS according to functional, proteinuric, and histological markers.

	ESRD no. 9 (24%)	Remission no. 23 (61%)
FE IgG 0 versus 1 (26 versus 12)	0% versus 75% (<0.0001)	77% versus 25% (0.016)
24 hP 0 versus 1 (20 versus 18)	0% versus 50% (0.001)	75% versus 44% (0.10)
UP/C 0 versus 1 (22 versus 16)	0% versus 56% (<0.0001)	73% versus 44% (0.11)
eGFR 0 versus 1 (20 versus 18)	30% versus 17% (0.23)	65% versus 56% (0.10)
FE *α*1m 0 versus 1 (28 versus 10)	7% versus 70% (<0.0001)	71% versus 30% (0.06)
*α*2m/C 0 versus 1 (26 versus 12)	4% versus 67% (<0.0001)	81% versus 17% (0.007)
FE IgG 0 + *α*2m/C 0 (23) versus FE IgG 1 + *α*2m/C 1 (9)	0% versus 89% (<0.0001)	83% versus 11% (0.008)
SS 0 versus 1 (17 versus 18)	6% versus 39% (0.018)	82% versus 44% (0.11)
GGS 0 versus 1 (21 versus 14)	24% versus 21% (0.80)	67% versus 57% (0.71)
TID score 0 versus 1 (22 versus 13)	27% versus 15% (0.39)	64% versus 62% (0.26)

FE IgG 0 versus 1: < versus ≥ 0.112; 24 hP 0 versus 1: < versus ≥ 6.8 g/24 hours; UP/C 0 versus 1: < versus ≥ 5980 mg/g uCr; eGFR 0 versus 1: < versus ≥ 68 mL/min/1.73 m^2^; FE *α*1m 0 versus 1: < versus ≥ 0.362; *α*2m/C 0 versus 1: < versus ≥ 4.79 mg/g uCr; SS 0 versus 1: < versus ≥ 16%; GGS 0 versus 1: < versus ≥ 7%; TID score 0 versus 1: < versus ≥ 3.
